# Conserved epigenetic hallmarks of T cell aging during immunity and malignancy

**DOI:** 10.1038/s43587-024-00649-5

**Published:** 2024-06-12

**Authors:** Tian Mi, Andrew G. Soerens, Shanta Alli, Tae Gun Kang, Anoop Babu Vasandan, Zhaoming Wang, Vaiva Vezys, Shunsuke Kimura, Ilaria Iacobucci, Stephen B. Baylin, Peter A. Jones, Christopher Hiner, April Mueller, Harris Goldstein, Charles G. Mullighan, Caitlin C. Zebley, David Masopust, Ben Youngblood

**Affiliations:** 1 Department of Immunology, St. Jude Children’s Research Hospital, Memphis, TN USA; 2 Center for Immunology, Department of Microbiology and Immunology, University of Minnesota, Minneapolis, MN USA; 3 Department of Computational Biology and Department of Epidemiology and Cancer Control, St. Jude Children’s Research Hospital, Memphis, TN USA; 4 Department of Pathology, St. Jude Children’s Research Hospital, Memphis, TN USA; 5 The Sidney Kimmel Comprehensive Cancer Institute, The Johns Hopkins Hospital, Baltimore, MD USA; 6 Department of Epigenetics, Van Andel Institute, Grand Rapids, MI USA; 7 Department of Microbiology and Immunology, Albert Einstein College of Medicine, New York, NY USA; 8 Department of Bone Marrow Transplantation and Cellular Therapy, St. Jude Children’s Research Hospital, Memphis, TN USA

**Keywords:** Lymphocytes, DNA methylation, Ageing

## Abstract

Chronological aging correlates with epigenetic modifications at specific loci, calibrated to species lifespan. Such ‘epigenetic clocks’ appear conserved among mammals, but whether they are cell autonomous and restricted by maximal organismal lifespan remains unknown. We used a multilifetime murine model of repeat vaccination and memory T cell transplantation to test whether epigenetic aging tracks with cellular replication and if such clocks continue ‘counting’ beyond species lifespan. Here we found that memory T cell epigenetic clocks tick independently of host age and continue through four lifetimes. Instead of recording chronological time, T cells recorded proliferative experience through modification of cell cycle regulatory genes. Applying this epigenetic profile across a range of human T cell contexts, we found that naive T cells appeared ‘young’ regardless of organism age, while in pediatric patients, T cell acute lymphoblastic leukemia appeared to have epigenetically aged for up to 200 years. Thus, T cell epigenetic clocks measure replicative history and can continue to accumulate well-beyond organismal lifespan.

## Abstract (web summary)

Using an iterative boost and transplantation model to generate multilifetime T cells, Mi et al. show that cellular epigenetic age can be uncoupled from organism age. While naive T cells appear epigenetically young, memory T cells and T-ALL leukemia can exhibit epigenetic ages exceeding the organismal lifespan.

## Main

Aging is often described as a path toward proliferative dysfunction, biasing the development of aging metrics toward a threshold that predicts maximal life, rather than simply tallying past experiences. Specifically, accumulation of age-associated changes in telomere length and somatic mutations have been reported as a surrogate for senescence and cellular lifespan^1–3^. Additionally, products of the *Cdkn2a*/*2b* gene cluster that regulate RB and p53 are elevated in somatic cells of aged mice, linking these genes with age-associated senescence^4–6^. Further supporting this link, it has been shown that conditional deletion of the gene encoding p16 (Ink4a) within this cluster in adult mice substantially prolongs their lifespan^7–9^. In humans, increased expression of senescence-associated gene products in somatic tissues and peripheral blood similarly correlates with organismal aging^1–4,10^. While these cell cycle regulatory principles can define a finite lifespan of somatic cells, memory T cells have an inherent ability to preserve telomere length during rapid and sequential rounds of antigen-driven replication^11–13^. The long-lived nature of memory T cells underscores conceptual and logistical challenges of using classic aging metrics to define chronologic immune age and raises the question of whether T cell experiential aging can supersede organismal lifespan.

Recent description of a multilifetime (ML) murine model of memory T cell differentiation demonstrated that T cells iteratively boosted and adoptively transferred over a 10 year period retained the ability to undergo antigen-driven proliferative bursts through 51 successive rounds of stimulation^13,14^. In other words, the daughter cells were able to evade replicative senescence without malignant transformation in a setting that far exceeded the organismal lifespan, highlighting the open question of whether cells that experience extraordinary longevity without malignant transformation do so by suspending the aging process. Given that functional memory T cells can live beyond organismal life without undergoing replicative senescence, we sought to determine if they possessed epigenetic metrics that correlated with the cell’s versus the host’s chronologic age. Recent attempts to define molecular clocks to predict organismal age have established epigenetic modifications as an age-estimator with remarkable accuracy^15–17^. Assessment of epigenetic chronology in hematopoietic stem cells has established a link between mitotic events and proliferative decline, thereby reinforcing the concept that epigenetic clocks measure a reduction in a cell’s functional potential^18^. However, these clocks have never been assessed in settings of cellular longevity that can exceed organismal lifespan^17^. In this Letter, we used the recently described in vivo ML murine model of memory T cell differentiation to identify epigenetic programs that correlated with the T cell’s proliferative history and longevity and demonstrate that epigenetic clocks can exceed an organismal lifespan without malignant transformation of the cell.

## Results

### T cell epigenetic remodeling proceeds beyond organismal lifespan

**Fig. 1:**
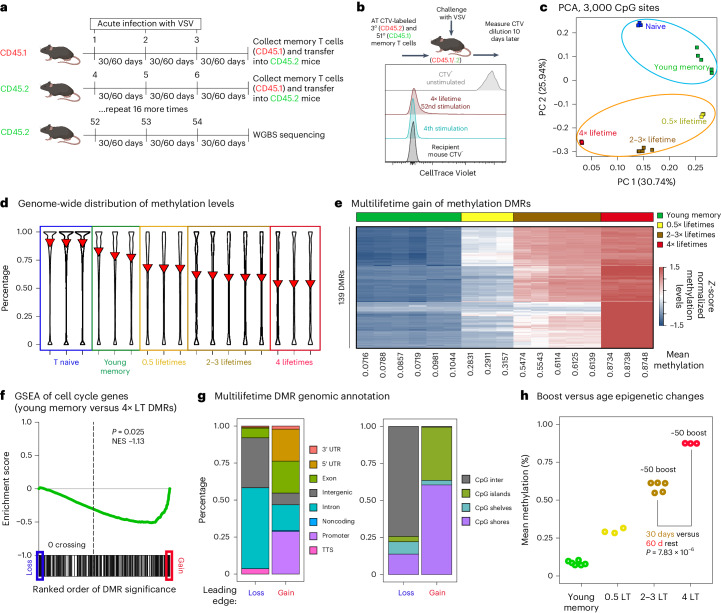
Epigenetic profiles of physiologically aged and ML functional memory T cells. **a**, Schema for generating ML memory CD8^+^ T cells. Naive mice were infected with VSV generating antigen-specific memory CD8^+^ T cells. Heterologous VSV infection and boosting of the memory T cells was performed after 30 versus 60 days post previous infection. After three infections, the antigen-specific memory CD8^+^ T cells were transferred into congenically distinct naive mice, and the boosting strategy was repeated over a time frame that yielded memory T cells with age ranging between 0.5 and ~4 mouse lifetime (LT) equivalents. For young memory, *n* = 6; 0.5× LT, *n* = 3; 2–3× LT, *n* = 5; and 4× LT, *n* = 3. **b**, Representative FACS analysis of cell trace violet (CTV) dilution from 3° (CD45.2) and 51° (CD45.1) memory T cells transferred into recipient CD45.1/2 mice. CTV label was measured 10 days after VSV-Indiana challenge of the chimeric mice. **c**, PCA plot of 3,000 most variable CpG sites comparing ML and ‘young’ memory T cells. Plot showing principal components 1 and 2 (PC1 and PC2). **d**, Violin plots showing average genome-wide CpG methylation for naive, young memory and ML CD8^+^ T cells. The red triangles represent the median values. **e**, Heatmap showing top ML gain of methylation DMRs. **f**, GSEA using cell cycle regulators and gain versus loss of methylation DMRs for 4× LT memory CD8^+^ T cells relative to young memory CD8^+^ T cells. The *P* value is based on the weighted Kolmogorov–Smirnov statistic with no adjustment. **g**, Genomic and CpG island annotation of top loss and gain of methylation ML DMRs. **h**, Summary graph showing mean methylation across top ML gain of methylation DMRs relative to boosting and age of memory T cells. Different lengths of resting period also result in significant changes of mean methylation levels. The *P* value is based on the two-sided Student’s *t*-test.

**Extended Data Fig. 1:**
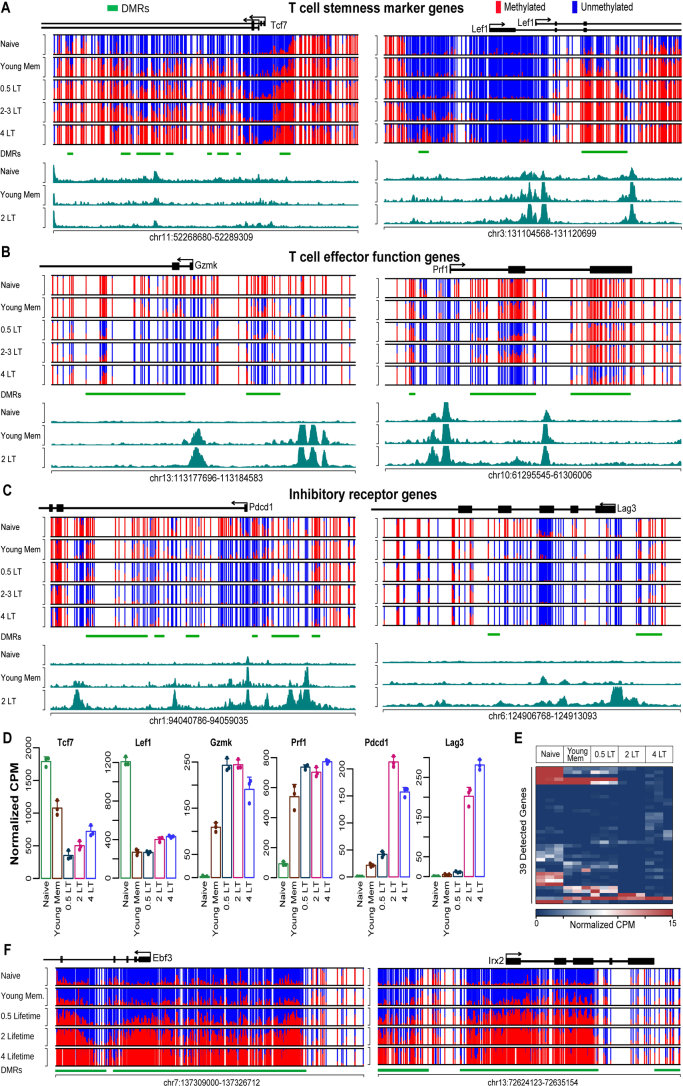
Transcriptomic and epigenetic profiles of multi-lifetime memory T cells at genes of interest. (**a**) DNA methylation level plots and ATAC seq tracks of genes associated with T cell stemness among multi-lifetime memory T cells. (**b**) Methylation level plots and ATAC seq tracks of genes associated with effector T cell functions among multi-lifetime memory T cells. (**c**) Methylation level plots and ATAC seq tracks of inhibitory receptor genes among multi-lifetime memory T cells. (**d**) Summary graphs for RNAseq transcript levels corresponding to genes in the methylation and ATACseq plots. n = 3 biologically independent samples. (**e**) Heatmap showing relative transcript abundance for cell-cycle regulators with measurable transcript defined from the ML memory T cell model. (**f**) Representative CpG DNA methylation of Ebf3 and Irx2 loci among top EA-associated DMRs from the multi-lifetime memory CD8 T cells.

To determine if epigenetic metrics of cellular aging transcend organismal lifespan, we employed an aging model in which CD8^+^ T cells far exceed the normal lifetime (LT) of the host^13^ (Fig. 1a). The mice were immunized with three heterologous prime–boost–boost immunizations (VSVnj, VVn and VSVind; Methods), with 30 versus 60 day intervals between infections. Congenically distinct VSV-specific CD8^+^ T cells were then adoptively transferred into naive mice that then underwent three additional immunizations with the heterologous virus schema. This adoptive transfer and boosting cycle was repeated up to 16 more times over 10 years to establish memory T cells ranging between 0.5 and ~4 mouse LTs (herein, 0.5 to 4× LT, based on an average lifespan of 2.5 years) relative to the original progenitor cells (Fig. 1a). The ML iteratively stimulated memory CD8^+^ T cells maintain a robust ability to proliferate throughout the 51-boost regimen^13^. Notably, the iteratively stimulated T cells preserved their proliferative capacity (Fig. 1b). Using purified ML memory T cells from this model we performed whole genome bisulfite sequencing to establish a nucleotide-resolution map of the DNA methylation changes that occur between physiologically aged and ML memory T cells. We first assessed the epigenetic relationship between young memory T cells and the ML memory T cells by performing a principal component analysis (PCA) of the top 3,000 most variable CpG sites (Fig. 1c). The results from this analysis revealed a distinct segregation between young memory CD8^+^ T cells versus the ML memory T cells, documenting CD8^+^ T cells’ ability to undergo further significant epigenetic modification throughout and far exceeding organismal lifespan. These results also indicate that analyses performed with young memory T cells fail to fully capture epigenetic programs associated with the later aging events (Fig. 1c). We next examined the genome-wide methylation distribution across T cells ranging from naive to 4 mouse LTs, with LT being defined as the chronologic time spanning from development of memory T cells established by the first round of prime–boost immunizations to the current boosted population of T cells. Notably, a significant reduction of global DNA methylation was observed between ‘young’ (~3 months post infection) memory T cells versus memory T cells analyzed at advanced age (~14 months that is, 0.5× LT) and multiple (>100 months, that is, 4× LT) organismal LTs (Fig. 1d). These findings extend previous observations concluding that aging is associated with a generalized decrease in DNA methylation^19^. Further interrogation of the DNA methylation profiles revealed exaggerated DNA demethylation of effector loci and inhibitory receptors that corresponded with ATAC sequencing-defined changes in chromatin accessibility and transcript expression (Extended Data Fig. 1a,d).

**Extended Data Fig. 2:**
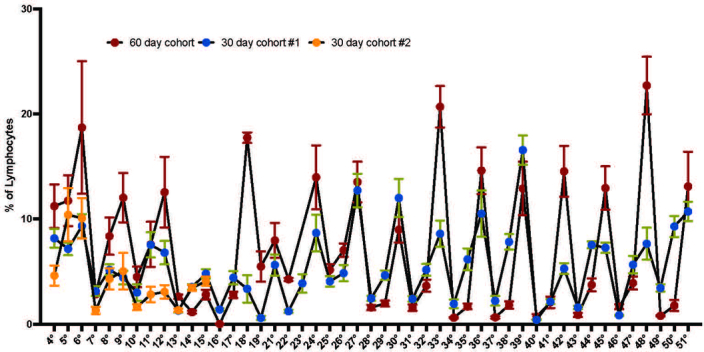
Summary graph showing the percentage of VSV-specific CD8 T cells generated from the heterologous prime-boost-boost infection schema throughout the entire time frame of the experiment. T cell frequency data from cohort 1 and 2 have been previously reported 19 and are shown here relative to the newly established cohort used to generate 0.5 LT memory CD8 T cells (cohort #3).

**Extended Data Fig. 3:**
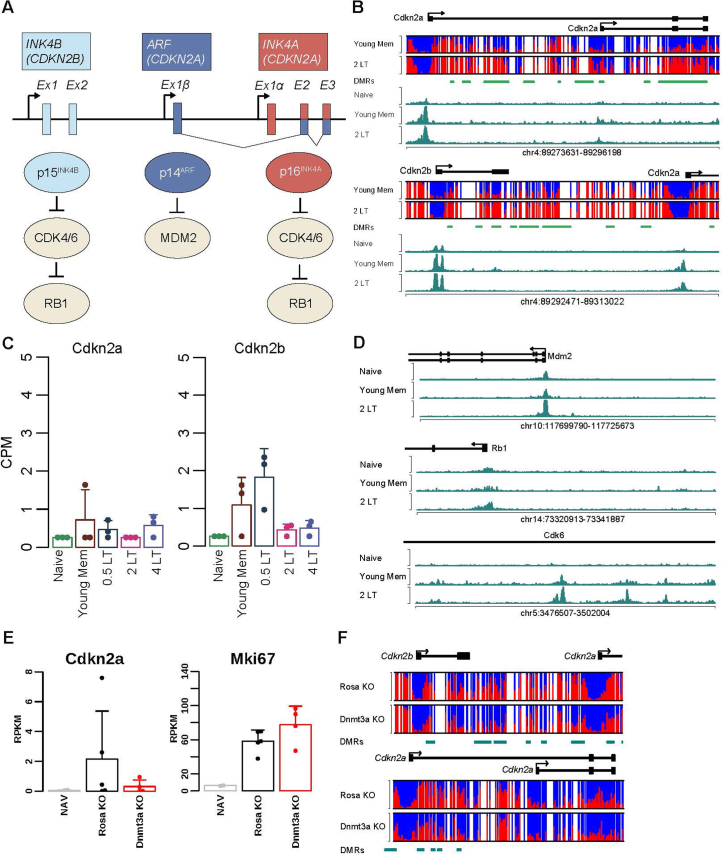
DNA methylation, chromatin accessibility and transcriptional profiles of p14/p16 cell cycle regulator genes. (**a**) Schematic cartoon of cell cycle regulator pathway related to p14/p16. Adapted from Sharpless and Sherr, Nature Reviews Cancer 2015. (**b**) DNA methylation and ATAC seq tracks showing the chromatin accessibility changes of DMR regions in the CDKN2A/2B locus. (**c**) Summary graphs for RNAseq transcript levels of CDKN2A and CDKN2B products. n = 3 biologically independent samples. Error bars represent standard deviation. (**d**) ATACseq profiles of Mdm2, Rb1 and Cdk6 loci. (**e**) Gene expression bar plots comparing Dnmt3a KO and Rosa KO (control). n = 2–5 biologically independent samples. Error bars represent standard deviation. (**f**) DNA methylation level plots showing Dnmt3a regulated gene body changes on Cdkn2a/2b.

Despite a generalized genome-wide reduction in DNA methylation, discrete regions of the genome became heavily methylated in an age-associated hierarchical manner with very little change in chromatin accessibility (Fig. 1e and Extended Data Fig. 1e,f). To further interrogate epigenetic modifications associated with cumulative age and experience despite retention of a T cell’s proliferative capacity, we performed various Gene Ontology (GO) and gene set enrichment analyses (GSEA). Differentially methylated regions (DMRs) between the young and ML memory T cells revealed significant enrichment of cell cycle-related genes among the gain of methylation DMRs (Fig. 1f). Genomic annotation of the GSEA leading edges revealed that gain of methylation DMRs were highly enriched for promoter, UTR and exonic regions relative to the loss of methylation DMRs (Fig. 1g). Several of these gain of methylation events occurred at genomic locations that were already inaccessible among naive T cells (Extended Data Fig. 3b,c). Furthermore, the gain of methylation was predominantly associated with CpG islands and shores (Fig. 1g), consistent with prior reports of age-associated changes in CpG island methylation^20,21^. Based on the genomic annotation results, we focused our attention on characterization of the top methylated regions among the iteratively boosted murine memory T cells (Supplementary Table 1). Quantification of the overall methylation for these regions compared to the relative LT of the samples revealed a temporal relationship that was dependent on the age of cells (Fig. 1h). We next sought to interrogate the effect of resting time between boosts and, thus, compared samples that rested for 60 or 30 days after a similar number of boosts. Notably, T cells boosted ~50 times with 60 day rests had a highly significant greater mean methylation among these DMRs as compared to T cells boosted ~50 times with only 30 days of rest (Fig. 1h). However, given that mouse memory T cells undergo a low rate of basal proliferation (often referred to as homeostatic self-renewal), this age-associated difference could be linked to cumulative proliferation. Additionally, the peak expansion of the boosted T cells was consistently much lower when the cells were only rested for 30 days, further indicating that ML memory T cells generated from 50 boosts with 30 days rest intervals underwent less proliferation relative to the cells rested for 60 days between boosts (Extended Data Fig. 2). Collectively, these results reveal that T cell aging results in the acquisition of DNA methylation at cell cycle regulator loci. Moreover, our ML model demonstrates that the epigenetic plasticity of T cells far exceeds organismal lifespan.

### Epigenetic modification of cell cycle regulators in aged T cells

**Fig. 2:**
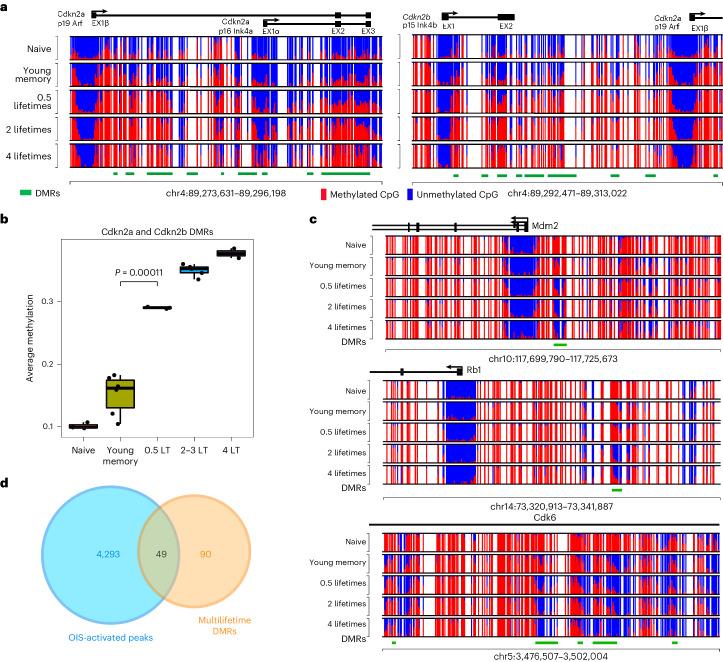
DNA methylation profiles of replicative senescence-associated genes. **a**, Representative CpG DNA methylation plot of *Cdkn2a*/*2b* loci in naive, young memory and ML memory CD8^+^ T cells. **b**, Summary graph of total DMR methylation among the Cdkn2a/b loci for young and ML memory CD8^+^ T cells. *n* = 2–5 for biologically independent samples. The *P* value based on a two-sided Student’s *t*-test. The box and hinges correspond to the first, second and third quartiles, the upper whisker extends to the minimum (largest value, upper hinge + 1.5 × interquartile range) and the lower whisker extends to the maximum (smallest value, lower hinge − 1.5 × interquartile range). **c**, Representative CpG DNA methylation of Mdm2, Rb1 and Cdk6. **d**, Venn diagram showing overlap between OIS-activated enhancers and top 139 DMRs.

Upon further examination, we noted that cyclin dependent kinase inhibitors (CDKs) *Cdkn2a* (p19^Arf^, p16^Ink4a^) and *Cdkn2b* (p15^Ink4b^) are among the genes enriched in the methylation signature (Fig. 2a and Extended Data Fig. 3a). The deletion or silencing of this gene cluster is commonly observed in various cancers, specifically malignancies of lymphoid origin^22–25^. A summary of the DMR methylation levels in the *Cdkn2a*/*b* loci shows a significant increase in average methylation levels from the young to ML memory CD8^+^ T cells (Fig. 2b). However, these DMRs are acquired in the gene body and exons, as opposed to the previously described CpG island methylation that represses expression in settings of malignancy^26^. Moreover, the notable absence of promoter methylation was accompanied by chromatin accessibility (Extended Data Fig. 3b). Such exonic and gene body methylation has historically been associated with transcriptional activity of these canonical senescence-associated genes^24^. Notably, gene expression from these loci was low in abundance in resting ML memory T cells (Extended Data Fig. 3c). We also examined the methylation status of other loci coding for proteins involved in regulating replicative senescence. Indeed, demethylation of the oncogenes *Mdm2*, *Rb1* and *Cdk6* (Fig. 2c) was inversely correlated with the gain of methylation observed at the *Cdkn2a*/*2b* gene cluster. Unlike the gain of methylation at the *Cdkn2a*/*2b* gene cluster, the decrease in DNA methylation of genes involved in tumor suppression was associated with an increase in chromatin accessibility (Extended Data Fig. 3d). To further interrogate the relationship between T cell proliferation and Cdkn2a gene body methylation, we analyzed expression of the gene products in *Dnmt3a*-knockout (KO) T cells that had experienced chronic stimulation. DNA methylation profiling confirmed that *Dnmt3a* regulates the gene body methylation of *Cdkn2a* and *Cdkna2b*. Importantly, *Cdkn2a* gene expression was higher in wildtype (Rosa irrelevant KO) T cells relative to *Dnmt3a* KO T cells, whereas *Mki67* gene expression (a surrogate for proliferation) was higher in *Dnmt3a* KO T cells (Extended Data Fig. 3e,f), consistent with our prior publication^27^.

To further assess our T cell age epigenetic signature for genes associated with replicative senescence, we next cross-referenced our DMR gene list with published oncogene-induced senescence (OIS)-specific regulatory elements^28^. Of the top gain of methylation DMRs observed in ML T cells, over one-third (49 genes) overlap with OIS-activated enhancers, many of which are cell cycle regulators (Fig. 2d). This enrichment further indicates that the gain of methylation program is coupled to a mechanism that enables T cells to avoid replicative senescence. Other significant genes of interest identified from the hypermethylated DMR list include those that encode additional cell cycle regulators, such as *Ebf3*, *Irx2* and *Sox1* (Extended Data Fig. 1f). Taken together, the epigenetic modification of genes encoding cell cycle regulators may regulate senescence of iteratively stimulated T cells and serve as a surrogate of T cell proliferation.

### T cell EA is uncoupled from chronologic age

**Fig. 3:**
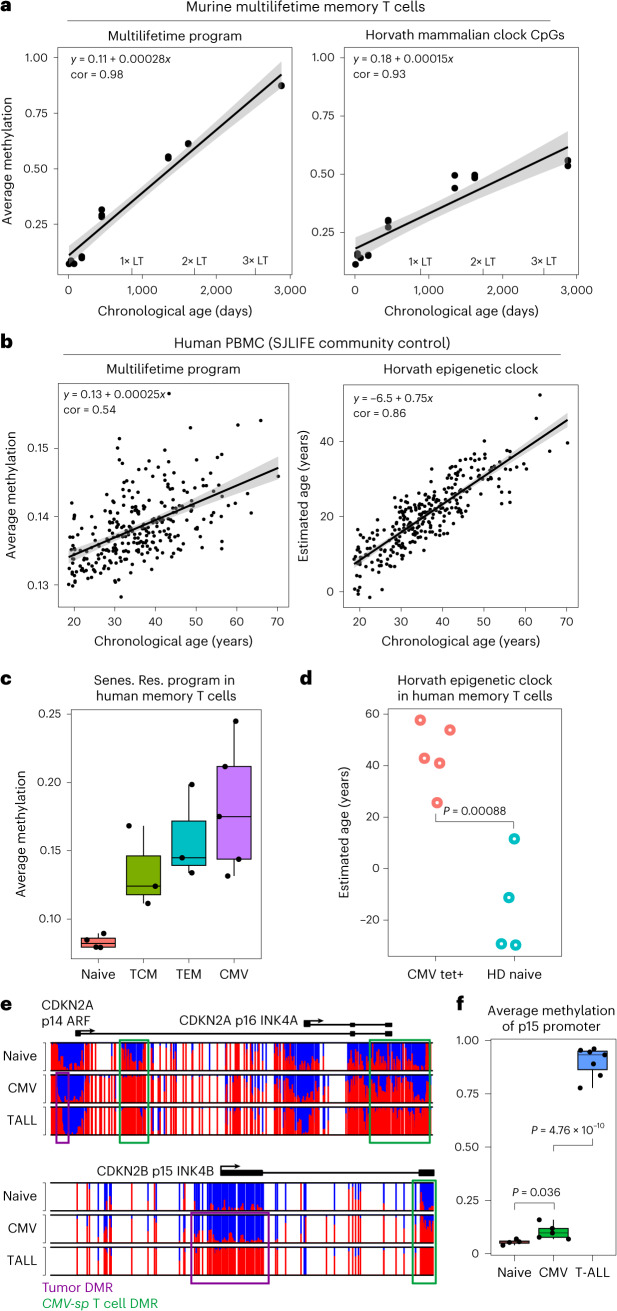
Age estimation of mouse and human immune cells using epigenetic clocks. **a**, Left: linear regression plots of mouse T cell age and average methylation levels of ML EA-associated genes. Right: linear regression plots of mouse T cell age and Horvath panmammal epigenettic clock. Pearson correlation coefficient (cor) is shown in each plot. The error band represents the mean ± 1.96 × standard error of the mean. **b**, Left: linear regression plots of SJLIFE healthy control (HC) chronologic age versus average PBMC methylation level. Right: linear regression plots of SJLIFE HC chronologic ages and Horvath epigenetic age estimation based on the PBMC methylation profile. Pearson cor is shown in each plot. The error band represents the mean ± 1.96 × standard error of the mean. **c**, Summary graph of average ML EA-associated methylation levels for human naive, Tcm, Tem and CMV-specific (Tetramer+) memory T cell samples. *n* = 3–5 for the biologically independent samples. Senescence resistant (Senes. Res.) program means the 139 top DMRs between endogenous and 4LT memory T cells. **d**, Horvath epigenetic clock age estimation of CMV-specific (*n* = 5) and naive (*n* = 4) CD8^+^ T cells. The *P* value is based on a two-sided Student’s *t*-test. The error bars represent the standard deviation. **e**, Representative DNA methylation plots of *CDKN2A/2B* gene cluster among naive CD8^+^ T cells, CMV-specific CD8^+^ T cells and T-ALL. **f**, Summary graph for p15 promoter methylation among naive CD8^+^ T cells, CMV-specific CD8^+^ T cells and T-ALL. *n* = 4–7 for the biologically independent samples. The *P* values are based on a two-sided Student’s *t*-test. For all box plots, the box and hinges correspond to the first, second and third quartiles, the upper whisker extends to the minimum (largest value, upper hinge + 1.5 × interquartile range) and the lower whisker extends to the maximum (smallest value, lower hinge − 1.5 × interquartile range).

**Extended Data Fig. 4:**
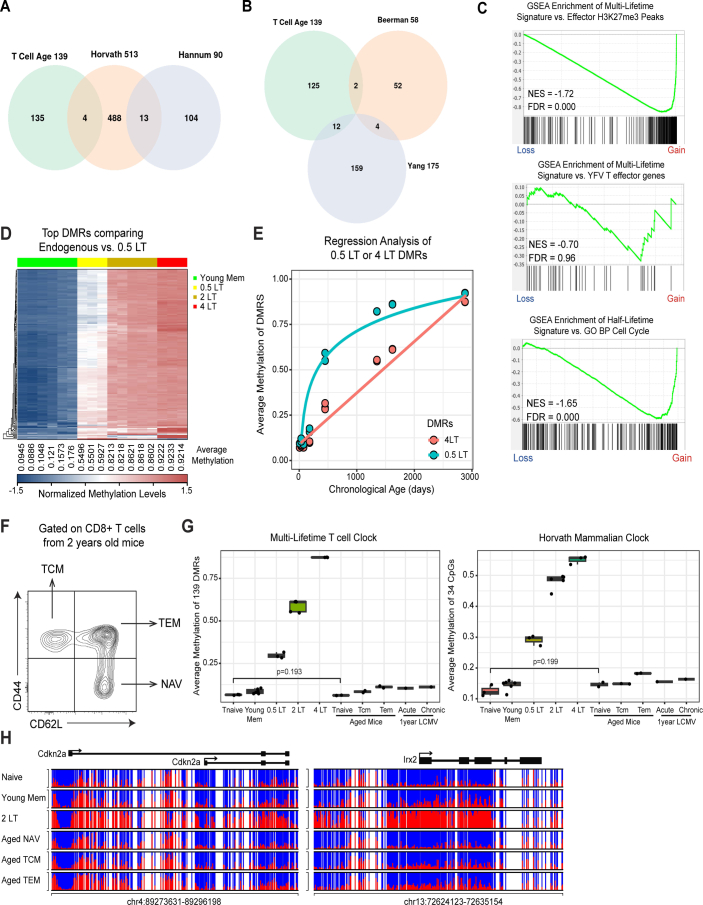
Annotation of multi-lifetime DMR genes. (**a**) Venn diagram of overlapped gene counts between the multi-lifetime EA program (T cell age), Horvath epigenetic clock and Hannum epigenetic clock. (**b**) Venn diagram of overlapped gene counts between T cell age, HSC aging hypermethylated genes 24 and EpiTOC mitotic age 39. (**c**) Top: GSEA enrichment of T Effector H3K27me3 peaks on 4 life-time defined DMRs. Middle: GSEA enrichment of T effector genes from YFV vaccine RNA seq data on 4 life-time defined DMRs. Bottom: GSEA enrichment of GO BP cell cycle genes on 0.5 life-time defined DMRs. All GSEA enrichment plots are comparable with main Fig. 1f. (**d**) Methylation heatmap of top 150 DMRs between endogenous and 0.5X lifetime samples. (**e**) Regression analysis of 0.5 LT DMRs versus 4X LT DMRs applied to all murine samples. (**f**) Representative FACS sorting of three T cell subsets in 2-year old mice cohort. (**g**) Mean methylation box plots of T cell age and Horvath mouse clock CpGs. n = 2–5 for multi-LT samples, n = 2 for aged mice samples, n = 1 for 1 year LCMV samples. P values based on two-sided Student’s t test.Box and hinges correspond to the first, second and third quartiles, upper whisker extends to min(largest value, upper hinge + 1.5 * IQR), lower whisker extends to max(smallest value, lower hinge - 1.5 * IQR). (**h**) Methylation level plots of Cdkn2a and Irx2 loci of T cells isolated from 2-year old mice.

Given that the progressive enrichment of DNA methylation at cell cycle regulatory genes was correlated to memory T cell age and proliferative history, we next sought to determine whether our ML DNA methylation signature was coupled to established epigenetic clocks. A linear regression analysis using T cells isolated from our murine ML model shows a positive correlation between the age of the cell and the average methylation of our core set of DMRs (Fig. 3a). Similarly, the Horvath clock^29^ also shows a positive correlation between the age of the murine T cells and the average methylation (Fig. 3a). Comparing the fit of these linear regression models shows that the mean methylation of the ML ‘experiential age’ (EA) program is better correlated with the chronologic age of the mouse T cell than the published epigenetic clock. Notably, there is little overlap between the CpG sites used for established epigenetic clocks and our EA program (Extended Data Fig. 4a). Established epigenetic clocks have been linked to the PRC2 complex^18,30^, yet we observed very little overlap with these genomic regions defined from nonimmune cell populations (Extended Data Fig. 4b). However, when we compared our ML programs to published effector T cell H3K27me3 modified genes^31^, we observed a striking enrichment among our ML programs (Extended Data Fig. 4c). Importantly, unlike prior observations made with stem cells^18^, our T cell-specific aging signature is not coupled to a decline in proliferation or effector potential. These data demonstrate that epigenetic clocks are not unequivocally linked to functional impairment.

We next asked if DMRs identified using 0.5× LT cells would track linearly with EA among the 2–4× LT samples. Notably, these programs plateaued at approximately one LT and are unable to accurately reflect EA beyond lifespan boundaries (Extended Data Figs. 4d,e). These data indicate that the putative clock derived from the ML model can measure a T cell’s proliferative history beyond organismal lifespan. To further test the hypothesis that T cell EA is autonomous of host chronologic age, we FACS (fluorescence-activated cell sorting) purified naive, central memory (Tcm) and effector memory (Tem) CD8^+^ T cell subsets from 2-year-old mice (Extended Data Figs. 4f and 8a,b) and performed whole genome methylation analysis. Global DMR analysis defined broad changes among the ML versus 2-year-old T cell subsets (Supplementary Table 2). Further analysis of overlapped DMRs between ML and memory T cells from 2-year-old mice, relative to naive T cells, identified conserved changes associated with memory T cell formation. GO analysis of the overlapping DMRs revealed enrichment of multiple lymphocyte activation/apoptosis-related pathways (Extended Data Fig. 9). Specific assessment of the ML EA T cell and the Horvath epigenetic clocks revealed that T cell subsets have similar ages in young and aged mice (Extended Data Fig. 4g,h). We next isolated 1-year-old LCMV (lymphocytic choriomeningitis virus)-specific T cells that had responded to a single infection. Similar to the 2-year-old polyclonal memory T cell subsets, the 1-year-old antigen-specific T cells had significantly reduced epigenetic aging signatures (Extended Data Fig. 4g,h). These data further indicate that epigenetic associated metrics of T cell aging are coupled to mitotic events rather than host age.

**Extended Data Fig. 5:**
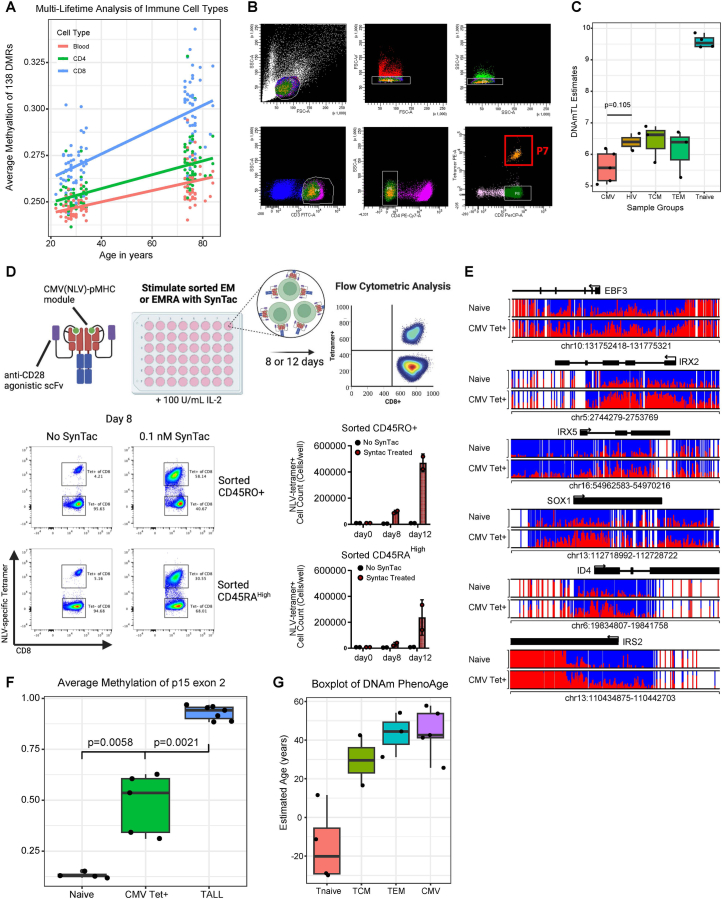
Characterization of multi-LT DMR genes among human CMV-specific T cells and reference methylation data sets. (**a**) T cells ages of 450K methylation array samples from matched donors (GSE59065). CD8 T cell samples showed strongest hypermethylation as the host age. (**b**) Representative FACS plot for isolating CMV Tet+ memory T cells. (**c**) Methylation based telomere length estimates of CMV-specific and HIV-specific T cells in matched donors. n = 2–5 biologically independent samples. P value based on two-sided Student’s t test. (**d**) Cartoon schematic and representative FACS plots showing tetramer staining among CD45RO+ and CD45RA+ CMV-specific T cells before and after in vitro expansion with SynTac. Bar plots show total number of expanded CMV-specific T cells from replicate experiments (n = 2). Error bars represent standard deviation. (**e**) DNA methylation level plots of EBF3, IRX2, IRX5, and SOX1 among naïve and CMV-specific CD8 T cells. (**f**) Summary graph for P15 exon 2 methylation among naïve CD8 T cells, CMV-specific CD8 T cells, and T-ALL. n = 5–7 biologically independent samples. P value based on two-sided Student’s t test. (**g**) Estimated Horvath DNAm PhenoAge for naïve, Tcm, Tem, and CMV-specific CD8 T cells. n = 3–5 biologically independent samples. For all box plots, box and hinges correspond to the first, second and third quartiles, upper whisker extends to min(largest value, upper hinge + 1.5 * IQR), lower whisker extends to max(smallest value, lower hinge - 1.5 * IQR).

We next sought to determine if the ML–EA program was conserved in human settings of T cell aging. We first utilized the St. Jude Life (SJLIFE) database of healthy individuals^32^ to perform a linear regression analysis between the age of the individual and the mean methylation of our ML DMRs among total PBMCs (peripheral blood mononuclear cells) (Fig. 3b). Indeed, there was a positive correlation between age of the individual and mean methylation of the top ML DMRs, consistent with the signal specifically coming from the T cell fraction of the total PBMC. Application of the Horvath clock^33^ yielded a stronger correlation between chronological age and estimated age, further confirming the ability of this published epigenetic clock to estimate age (Fig. 3b). However, despite the positive correlation, the Horvath clock appeared to underestimate the host age, suggesting that immune cells may age differently than the organism at-large. We next applied our ML–EA epigenetic clock to CD4^+^ and CD8^+^ T cell DNA methylation data collected from young and aged people^34^. Notably, we observed a greater age-associated increase in the average methylation among the CD8^+^ T cells (Extended Data Fig. 5a).

**Extended Data Fig. 6:**
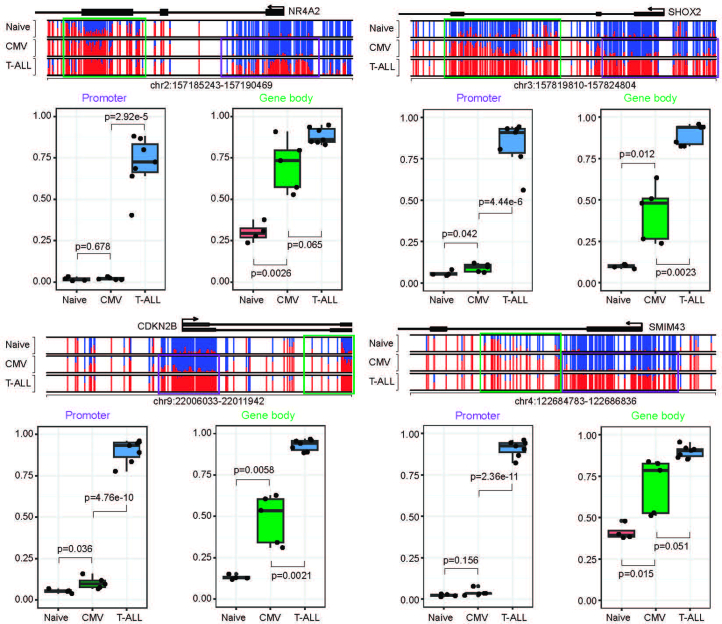
Leukemia associated vs. proliferation associated DMRs. Top panels: Representative CpG DNA methylation of NR4A2, CDKN2B, SHOX2, and SMIM43 leukemia-associated DMRs. Corresponding summary graph of individual loci promoter and gene body CpG island methylation among naïve CD8 T cells, CMV-specific CD8 T cells, and T-ALL. n = 4–7 biologically independent samples. P values based on two-sided Student’s t test. Box and hinges correspond to the first, second and third quartiles, upper whisker extends to min(largest value, upper hinge + 1.5 * IQR), lower whisker extends to max(smallest value, lower hinge - 1.5 * IQR). Bottom panels: Representative CpG DNA methylation of Nr4a2 comparing iteratively stimulated murine ML T cells versus WT and DNMT3A KO P14 CD8 T cells from mice chronically infected with LCMV for 30 days.

To further assess age estimates from the established epigenetic clock and our ML–EA program, we focused our analysis on specific human T cell populations with known developmental states and infection history. We first evaluated the ML–EA program in well-defined human CD8^+^ T cell subsets from healthy adults^35,36^. Notably, Tcm and Tem CD8^+^ T cells were significantly enriched for the ML–EA program relative to naive CD8^+^ T cells from the same donors (Fig. 3c). To further assess the relationship between the ML–EA program and T cell age, we isolated cytomegalovirus (CMV) specific memory CD8^+^ T cells from 50–60-year-old individuals. Based on their inflationary phenotype, these CMV-specific CD8^+^ T cells reflect a human setting of repeated exposure to antigen over long periods of time (Extended Data Fig. 5b). Accordingly, the CMV-specific memory CD8^+^ T cells had the highest levels of ML–EA-associated methylation, with telomere length estimated to be comparable to other memory T cell subsets. (Fig. 3c and Extended Data Fig. 5c,g,e). Similarly, the Horvath epigenetic clock estimated that the CMV-specific CD8^+^ T cells to have an age ranging between 20 and 50 years old, whereas the naive CD8^+^ T cells had age estimates around 0 years (Fig. 3d). Similar to the murine ML CD8^+^ T cells, CMV-specific CD8^+^ T cells still retained a capacity to proliferate (Extended Data Fig. 5d) and did not exhibit signs of malignant transformation. Given the prior association between *CDKN2A*/*2B* promoter silencing with malignant transformation, we sought to compare the methylation status of the locus in CMV-specific T cells versus leukemia. While both T cell populations have methylated the gene body and exons of this locus, only the leukemia acquired methylation at the promoter region (Fig. 3e,f, Extended Data Figs. 5f and 6 and Supplementary Tables 3 and 4). These data suggest that ML–EA-associated DMRs (Fig. 3e) track T cell proliferation irrespective of host age and are not classical malignancy programs.

### Human leukemias undergo accelerated epigenetic aging

**Fig. 4:**
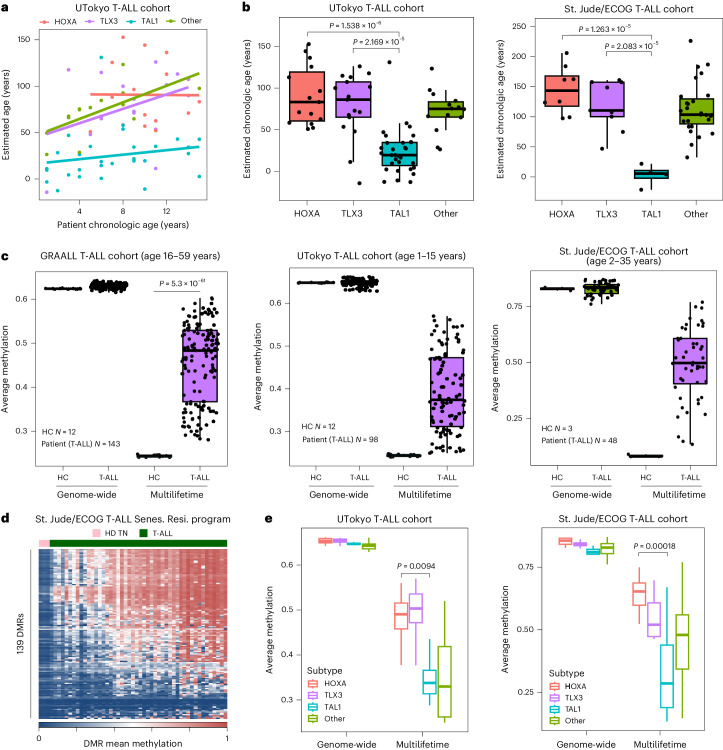
T-ALL subset epigenetic age estimation. **a**,**b**, Summary graphs of patient chronologic age (**a**) versus estimated tumor age (**b**) based on T-ALL subset using the Horvath epigenetic clock (*n* = 13–31 in the UTokyo cohort and *n* = 4–28 in the St. Jude cohort). The *P* values are based on a two-sided Student’s *t*-test. **c**, Summary box plots for the T-ALL patient tumor genome-wide DNA methylation levels versus EA programs among three independent cohorts (GRAALL *n* = 143, St. Jude/ECOG *n* = 48 and UTokyo *n* = 98). The *P* value is based on a two-sided Student’s *t*-test. **d**, DNA methylation level heatmap of ML EA programs for St. Jude/ECOG T-ALL patients. **e**, Summary graph of ML EA programs among established T-ALL patient subtypes. The *P* values are based on two-sided Student’s *t*-tests comparing *HOXA* and *TAL1* subtypes (*n* = 13–31 in the UTokyo cohort and *n* = 4–28 in the St. Jude cohort. For all box plots, the box and hinges correspond to the first, second and third quartiles, the upper whisker extends to the minimum (largest value, upper hinge + 1.5 × interquartile range) and the lower whisker extends to the maximum (smallest value, lower hinge − 1.5 × interquartile range).

**Extended Data Fig. 7:**
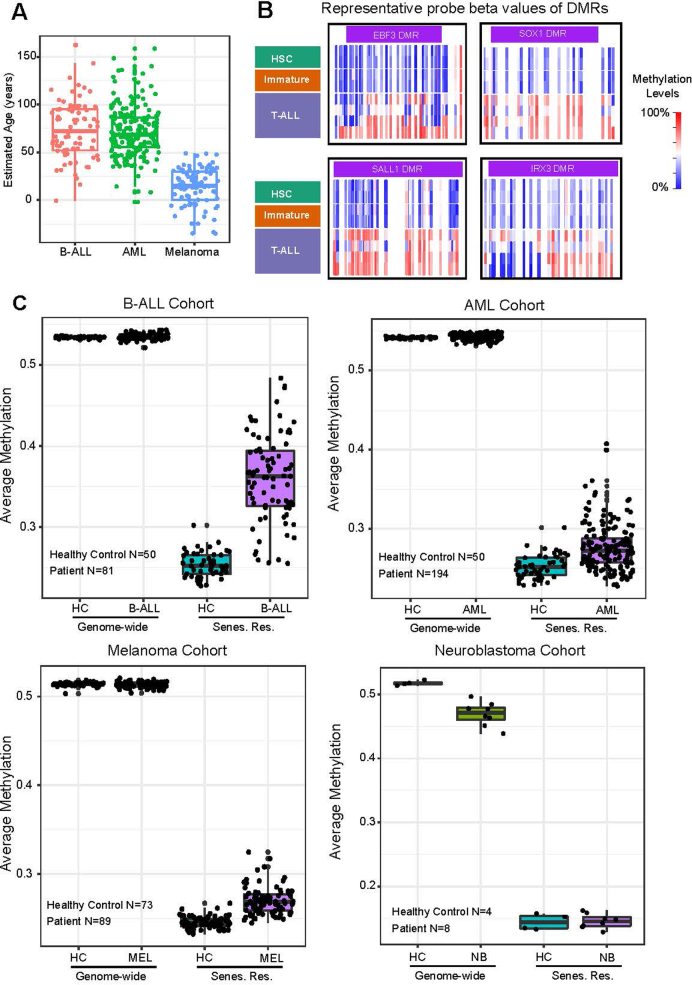
DNA methylation pattern of multi-LT DMRs among various human cancer datasets. (**a**) Epigenetic age estimation of four cancer patient cohorts based on Horvath DNAm PhenoAge. n = 81 for B-ALL samples, n = 194 for AML samples, n = 89 for melanoma samples. (**b**) DNA methylation level plots on selected EA genes. (**c**) Average methylation box plot comparing genome wide methylation versus EA program among healthy controls and cancer patients with B-ALL, AML, melanoma or neuroblastoma. For all box plots, box and hinges correspond to the first, second and third quartiles, upper whisker extends to min(largest value, upper hinge + 1.5 * IQR), lower whisker extends to max(smallest value, lower hinge - 1.5 * IQR).

**Extended Data Fig. 8:**
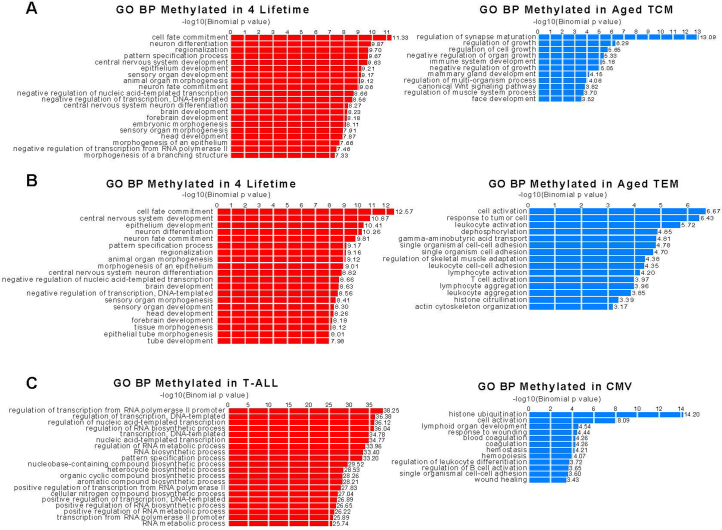
Gene ontology annotation of DMRs from mouse and human aged T cell comparisons. (**a**) Top GO enriched terms for aged mice Tcm vs 4 Lifetime samples. (**b**) Top GO enriched terms for aged mice Tem vs 4 Lifetime samples. (**c**) Top GO enriched terms for T-ALL vs. CMV-specific T cells. P values based on one-sided binomial test with no adjustment.

**Extended Data Fig. 9:**
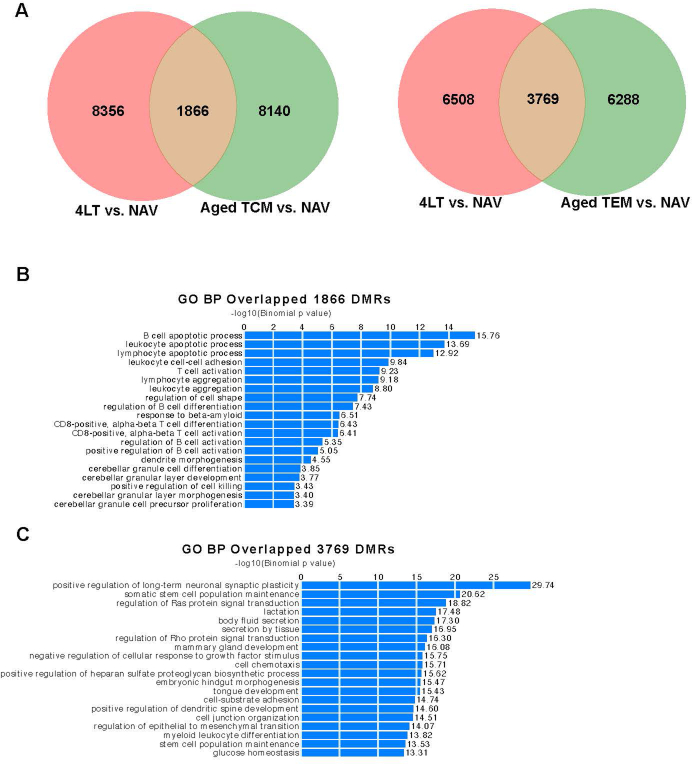
Overlap analysis of multi-LT DMRs vs. aged mice DMRs. (**a**) Venn diagrams of overlaps between 4LT defined DMRs and 2 year old aged mice defined DMRs. (**b**) Top GO enriched terms of overlapped DMRs between 4LT and aged TCM DMRs. (**c**) Top GO enriched terms of overlapped DMRs between 4LT and aged TEM DMRs. P values based on one-sided binomial test with no adjustment.

We next expanded our interrogation of the T cell experiential aging signatures by evaluating it in publicly available DNA methylation profiles from various hematological and solid tumor cohorts^37–41^. We first used the Horvath clock to establish age estimates using DNA methylation profiles from patients with T cell acute lymphoblastic leukemia (T-ALL), B cell acute lymphoblastic leukemia (B-ALL), acute myeloid leukemia (AML) and melanoma (Fig. 4a,b and Extended Data Fig. 7a). Notably, hematologic malignancy DNA methylation profiles yielded a significantly greater age estimation than observed with melanoma. We next proceeded to determine if T-ALL subtypes were specifically linked to different age estimates. Using a pediatric T-ALL cohort with patient ages ranging between 1 and 15 years old, we compared the patients’ chronologic age and the estimated age. Quite strikingly, several of the adolescent patients had leukemias that were estimated to be >100 years old. Furthermore, we noted that the malignancies with an exaggerated age prediction, including *HOXA* and *TLX3*, were derived from transformation events that occurred earlier in T cell development (Fig. 4a,b). Given the current clinical need for novel metrics to delineate T-ALL subtypes, we proceeded to validate the early T cell precursor subtype using DNA methylation profiles obtained from an independent cohort at St Jude/ECOG (Eastern Cooperative Oncology Group). Again, we observed a remarkable dichotomy in age estimates among the *HOXA* and *TLX3* subtypes versus the *TAL1* subtypes, revealing leukemias derived from early developmental stages to have age estimates ranging from ~100–200 years old. Thus, well-established epigenetic clocks predict T-ALL subtypes to exhibit hallmarks of accelerated experiential aging exceeding human lifespan.

We next interrogated the various cancer cohorts using our murine ML–EA methylation signature (Fig. 4c and Extended Data Fig. 7b,c). Comparison of the genome-wide average level of methylation between healthy controls versus the malignancy cohorts revealed that the ML–EA program was significantly enriched in the patients with hematological malignancies (Fig. 4c and Extended Data Fig. 7b,c). Notably, T-ALL patients had the greatest enrichment of the ML–EA program relative to the B-ALL and AML-derived samples (Fig. 4c and Extended Data Fig. 7b,c). The robust enrichment of the ML–EA program among lymphocytic leukemias further documents a link between a T cells’ putative proliferative history and epigenetic repression of cell cycle regulators.

While there was indeed significant enrichment of the ML–EA program among all three independent T-ALL cohorts, each cohort possessed a striking degree of heterogeneity in the relative level of the signature (Fig. 4c,d). To further interrogate this heterogeneity, we analyzed the ML–EA program based on the known T-ALL subtypes. Similar to the age estimate, our ML–EA program was most enriched among T-ALL derived early in T cell development, namely leukemias with *HOXA* and *TLX3* genetic mutations (Fig. 4e). These data collectively document that the ML–EA methylation programs can delineate human T-ALL subtypes based on T cell maturation stage and that the accumulation of epigenetic aging metrics is uncoupled from replicative senescence.

## Discussion

Our results here document epigenetic modifications coupled to the proliferative history of T cells and highlight that progressive enrichment of gene body methylation occurs at genes known to control cell cycle entry and accumulate without evidence of replicative senescence. Thus, epigenetic clocks are not bound by organismal lifespan and do not explicitly countdown to replicative dysfunction. The relationship between human chronologic age and T cell epigenetics has been previously described by several groups^42^, documenting an enrichment of memory-associated epigenetic programs in aged populations. Our results extend these observations by describing a specific epigenetic program that is uncoupled from human memory T cell proliferative decline as well as a decline in naive T cell output due to thymic involution. Taken together, these data are consistent with T cell epigenetic clocks functioning autonomously from organismal age.

Our use of a ML murine model of memory T cell differentiation to identify DNA methylation programs linked to proliferative history enabled us to define experience-specific molecular features that were not readily observed in traditional murine infection models, and now link ‘epigenetic clocks’ to a discrete biological process. Moreover, our results demonstrate that epigenetic clocks continue to track cellular age beyond organismal lifespan boundaries indicating that the aging process is not absolutely bounded by a finite time frame. However, given that immune senescence is a prevailing outcome as we age, our data further highlight a critical role for cell-extrinsic factors such as senescence-associated secretory phenotype-derived proteins^43^ in driving the decline in immunological protection.

Despite having a chronologic age of approximately four organismal LTs, there is no evidence of malignant outgrowth identified in the iteratively stimulated murine memory T cells. The ability of these T cells to undergo sustained but controlled proliferation further supports the previously described notion that mature T cells are strikingly resistant to malignant transformation^13^. Thus, while there is a general association with hypermethylation and risk for malignant transformation^44,45^, our results highlight the specific exclusion of promoters, including p15 and p16, from the ML-associated hypermethylation profile and broadly illustrate the distinction in genomic location of epigenetic events that delineate transformation versus longevity. Though further experiments are needed to establish a causal relationship between these promoter methylation programs and T cell senescence or malignancy, these results collectively highlight a need to better define the malignancy checkpoints that protect functional memory T cells from transformation. While our data do not directly test for selective enrichment of a functional T cell clone, it is conceivable that the repetitive nature of the murine iterative transplantation model system would exert a selective pressure for the specific survival of a discreet T cell subset. With further advancement in our understanding of T cell epigenetic aging metrics, we can begin to rationalize therapeutic approaches for halting or reversing age-associated impairments. In summary, discovery of a T cell epigenetic clock that is uncoupled from proliferative decline highlights T cell aging metrics that are distinct from the properties that limit somatic cell lifespan and provide a hallmark for assessing cancer-free T cell aging.

## Methods

### Mice and Infection

Donor female B6.SJL-Ptprc^a^Pepc^b^/BoyJ (CD45.1^+^ B6, around 8 weeks old) were bred at the University of Minnesota animal facilities. Female C57BL/6J (CD45.2^+^ B6, around 8 weeks old) mice were purchased from Jackson Laboratories and served as recipient mice. Aged 2-year-old mice were purchased from Jackson Laboratories. The animals were treated according to the University of Minnesota and St. Jude Children’s Research Hospital Institutional Animal Care and Use Committee guidelines. CD45.1^+^ tertiary memory cells were expanded through heterologous prime–boost–boost infection with 10^6^ PFU vesicular stomatitis virus (VSV) subtype New Jersey (NJ), an experiment-specific period of rest, 2 × 10^6^ PFU vaccinia virus (VV) expressing the VSV-Indiana nucleoprotein (N), an experiment-specific period of rest, and 10^7^ PFU of VSV-Indiana upon transfer to recipient CD45.2^+^ mice, cells were expanded through heterologous prime–boost–boost infection with 10^6^ PFU of VSV-Indiana, an experiment-specific period of rest, 2 × 10^6^ PFU VV-N, an experiment-specific period of rest and 10^7^ PFU VSV-NJ. All heterologous prime–boost–boost infections were delivered via the tail vein.

### Mouse T cell isolation and adoptive transfer

A single cell suspension was prepared from the spleen and macroscopic lymph nodes of donor mice. CD8^+^ T cells were enriched via negative selection before they were stained with surface antibodies. CD8^+^ T cell enrichment was done using the Stem Cell EasySep Mouse CD8^+^ T Cell Isolation Kit following manufacturer’s instructions or using a prepared cocktail of biotinylated antibodies. The purified CD8^+^ T cells were then stained with anti-mouse CD8a (53-6.7) from BD Biosciences, anti-mouse CD45.1 (A20), anti-mouse CD45.2 (104), Ghost Dye from Tonbo Biosciences and N-Tetramer. CD8a^+^, VSV-N-Tetramer^+^, CD45.1^+^ and CD45.2^−^ cells were sorted on a BD FACS Aria II, and 10^5^ sorted cells were transferred via the tail vein into recipient mice. Infections resumed the following day. FlowJo v10 software was used to visualize flow cytometry analysis.

### Chronic stimulation of LCMV-specific T cells

Naive CD8^+^ T cells were isolated from spleens of P14 mice, following manufacturer’s instructions (Stem Cell EasySep). The CD8^+^ T cells were activated in the presence of 0.5 µg ml^−1^anti-CD3 and 5 µg ml^−1^anti-CD28 antibody (Thermo Fisher). RNP complexes were formed for 20 min at room temperature using Cas9 (Alt-R S.p. Cas9 Nuclease, IDT) and sgRNA per target (Synthego, IDT). Electroporation was performed on 10^6^ CD8^+^ T cells using 4D-Nuvleotector X Unit and P4 primary cell 4D-Nucleofector TM X Kit, with pulse CM137 (Lonza). The cells were rested at 37 °C for 3–4 days. The cells were collected and washed with phosphate-buffered saline before transfer in recipient mice. The sequences of sgRNA used to target: *Rosa gRNA*: GACTCGAGTTAGGCCCAACG*, Dnmt3a gRNA1*: GATCATTGATGAGCGCACAA*, Dnmt3a gRNA2*: CTTACCAGTATGACGACGAT and *Dnmt3a gRNA3*: *CAGGCCGAATTGTGTCTTGG*. After 3–4 days post electroporation with RNP, 10^4^ P14 cells were injected into recipient mice 18–24 h before infection. Chimeric mice were infected with LCMV clone 13 (2 × 10^6^ PFU, intravenous). The P14 T cells were isolated from the chronically infected mice after 30 days or 1 year (P14 cells obtained from acute and chronically infected mice) and processed for whole genome methylation profiling and RNA sequencing.

### Human T cells

CMV-specific CD8^+^ T cells were FACS purified from donor blood samples obtained by leukapheresis using an institutional review board-approved protocol of the Einstein–Rockefeller–CUNY Center for AIDS Research. Written informed consent was obtained from donors according to institutional review board requirements. The PBMCs were isolated from deidentified blood samples using Ficoll density gradient centrifugation, washed, resuspended in in freezing media (10% dimethylsulfoxide in fetal bovine serum), aliquoted (5 × 10^7^ cells ml^−1^) and stored at −150 °C. The freshly thawed PBMCs from human immunodeficiency virus (HIV) seropositive or HIV seronegative donors were washed with FACS buffer (phosphate-buffered saline containing 5% fetal bovine serum and 1 mg ml^−1^sodium azide) and 4 × 10^6^ PBMCs were transferred per well in a 96-well plate. Each well was incubated with 1 µl human FcR Blocking Reagent (Miltenyi Biotec) in 50 µl FACS buffer for 15 min at room temperature. After the PBMCs were stained with 0.2 µl NLV-tetramer (NIH Tetramer Core Facility) (CMV pp65; amino acids 495NLVPMVATV503) in 60 µl per well for 25 min at room temperature, the PBMCs were incubated with 1.4 µl anti-CD3 FITC (BD Pharmingen, cat. 555339), 2.8 µl anti-CD4 PE/cy7 (BioLegend, cat. 300512) and 2.8 µl anti-CD8 PerCP-cy5.5 (BioLegend, cat. 344710) in 70 µl per well for 30 min at 4 °C. The PBMCs were washed and the CD3^+^, CD4^−^, CD8^+^, NLV-tetramer^+^ single cells were isolated by flow cytometric sorting using a FACS Aria II (Becton Dickinson). The DNA was extracted from the sorted cells using the DNeasy Blood and Tissue kit (QIAGEN) according to the manufacturer’s protocol.

### CMV-specific T cell proliferation assay

Sorted CD45RO^+^ (Tem) or CD45RO^−^ CD45RA^High^ (Temra) cells were stimulated with 0.1 nM NLV-antiCD28 SynTac^46^ and were stained and analyzed on days 8 and 12 following stimulation. NLV-specific CD8^+^ T cells accumulation was assessed as normalized cell count ((cell count μl^−^sample acquired on cytometer) × total sample volume). The mean was displayed with the standard deviaiton of technical duplicates using one donor PBMC in a single independent experiment.

### Whole genome DNA methylation profiling

DNA libraries were sequenced using Illumina NovaSeq 6000 systems. Reads have been trimmed by 10 bp on 5′ and 3′ ends as well as all Illumina adapter sequences. The trimmed reads were aligned to the mm10 genome using the BSMAP v. 2.90 software^47^.

### Differential methylation analysis and PCA

Differential methylation analysis was done by R package DSS 2.34^48^. Basic two group comparisons were ran, and a *P* value threshold of 0.01 was used. DMR results with top 150 largest absolute area statistics were used for further pathway analysis. A PCA was carried on top 3,000 CpG sites with the highest variance. Only sites with higher than five reads coverage in all samples were included. The R function princomp was used to compute components.

### GO pathway enrichment and GSEA

GO annotation was carried using GREAT web server (https://great.stanford.edu/great/public/html/). The input regions include the top 150 methylated and demethylated DMRs. The bar graph is based on top enriched GO Biological Process pathways. Preranked GSEA^49^ analysis was performed using DMR tables. The ranks for all genes were assigned by multiplying sign of log fold change and negative log10 of the *P* values. Only one DMR with largest absolute rank were used to represent a gene. The cell cycle gene signature is selected from GOBP_CELL_CYCLE gene set in MSigDB.

### Age estimation of WGBS and methylation array samples

Epigenetic ages of human samples were estimated based on DNAm PhenoAge paper^33^. The methylation beta values of the 513 CpG sites were calculated using methratio.py script of BSMAP for whole genome bisulfite sequencing (WGBS) samples and getBeta function of minfi R package^50^ for methylation array samples. The beta values were then multiplied with published weights and added the intercept to form age estimates. For mouse samples, epigenetic ages were estimated based on preprint data^51^. The phenotypic age estimates not only track with chronological age but also represent mortality and morbidity risk. Thus, their values can be negative. A total of 35 selected CpG sites that are hypermethylated in aged individuals were used. The methylation beta values were calculated similar to human samples. The average beta values of these 35 CpGs were used to represent mouse epigenetic ages.

### Statistics and reproducibility

No statistical method was used to predetermine sample size, but our sample sizes are similar to those reported in previous publications^13,27,35^. No data were excluded from the analyses. The experiments were not randomized. The investigators were not blinded to allocation during experiments and outcome assessment. Data distribution was assumed to be normal, but this was not formally tested. The statistical tests used in this study include weighted Kolmogorov–Smirnov test for GSEA, one-sided binomial test with no adjustment for GO enrichment and two-sided Student’s *t*-tests for all other comparisons. The numbers of biological replicates can be found in each figure legends.

### Publicly available human cancer DNA methylation datasets

GRAALL Epic array data of T-ALL patients were downloaded from the Gene Expression Omnibus (GEO) with accession number GSE147667 ref. ^37^. The University of Tokyo Epic array data of T-ALL patients were downloaded from National Bioscience Database Center database with accession number JGAS000138 ref. ^38^. 450K methylation array data of B-ALL patients were downloaded from the GEO database with accession number GSE49031 ref. ^39^. 450K methylation array data of patients with AML were downloaded from The Cancer Genome Atlas (TCGA) data portal with project ID TCGA-LAML^40^. 450K methylation array data of patients with melanoma were downloaded from the GEO database with accession number GSE120878 ref. ^41^. 450K methylation array data of patients with neuroblastoma were downloaded from the GEO database with GSE54719 ref. ^52^. All methylation array samples were process by Bioconductor workflow based on minfi R package. The samples were normalized with the preprocess Quantile function.

### Reporting summary

Further information on research design is available in the Nature Portfolio Reporting Summary linked to this article.

### Supplementary information

Reporting Summary

Supplementary Table Supplementary Tables 1–4.

## Figures and Tables

**Extended Data Fig. 10:**
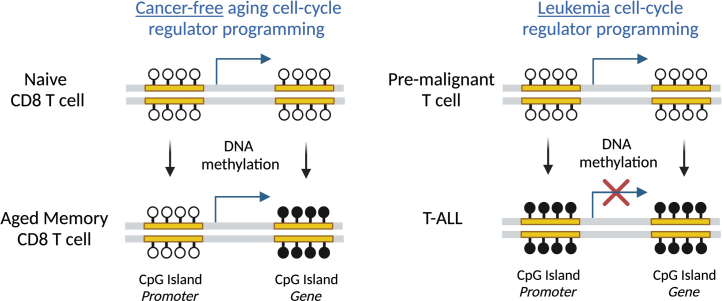
Cartoon summary of experiential age-associated DNA methylation programs that delineate longevity versus malignancy among T cells.

## Data Availability

Whole genome bisulfite sequencing data was deposited to GEO database under accession number GSE263941. Publicly available methylation data were downloaded from GEO with accession numbers GSE147667, GSE49031, GSE120878 and GSE54719. AML methylation data were downloaded from the TCGA data portal with project ID TCGA-LAML. Utokyo T-ALL methylation data were downloaded from National Bioscience Database Center database with accession number JGAS000138. Custom R scripts used for WGBS and methylation array analysis are available upon request. All other data supporting this study are available from the corresponding authors.
