# Supplementary material for: Conserved epigenetic hallmarks of T cell aging during immunity and malignancy
Source: Nat Aging. 2024 Jun 12;4(8):1053–63. doi: 10.1038/s43587-024-00649-5 (PMC11333289; doi:10.1038/s43587-024-00649-5)
Supplement: Supplementary file 1 — Reporting Summary [file 43587_2024_649_MOESM1_ESM.pdf]

nature portfolio

Corresponding author(s): Benjamin Youngblood, Caitlin Zebley, David Masopust

Last updated by author(s): Apr 22, 2024

Reporting Summary

Nature Portfolio wishes to improve the reproducibility of the work that we publish. This form provides structure for consistency and transparency in reporting. For further information on Nature Portfolio policies, see our Editorial Policies and the Editorial Policy Checklist.

Statistics

For all statistical analyses, confirm that the following items are present in the figure legend, table legend, main text, or Methods section.

n/a	Confirmed	
☐	☒	The exact sample size (*n*) for each experimental group/condition, given as a discrete number and unit of measurement
☐	☒	A statement on whether measurements were taken from distinct samples or whether the same sample was measured repeatedly
☐	☒	The statistical test(s) used AND whether they are one- or two-sided<br>*Only common tests should be described solely by name; describe more complex techniques in the Methods section.*
☒	☐	A description of all covariates tested
☐	☒	A description of any assumptions or corrections, such as tests of normality and adjustment for multiple comparisons
☐	☒	A full description of the statistical parameters including central tendency (e.g. means) or other basic estimates (e.g. regression coefficient) AND variation (e.g. standard deviation) or associated estimates of uncertainty (e.g. confidence intervals)
☐	☒	For null hypothesis testing, the test statistic (e.g. *F*, *t*, *r*) with confidence intervals, effect sizes, degrees of freedom and *P* value noted<br>*Give P values as exact values whenever suitable.*
☒	☐	For Bayesian analysis, information on the choice of priors and Markov chain Monte Carlo settings
☒	☐	For hierarchical and complex designs, identification of the appropriate level for tests and full reporting of outcomes
☐	☒	Estimates of effect sizes (e.g. Cohen's *d*, Pearson's *r*), indicating how they were calculated

Our web collection on statistics for biologists contains articles on many of the points above.

Software and code

Policy information about availability of computer code

Data collection	BD FACS Aria III, BD FACS Canto II, BD FACS Lyric, BD LSRFortessa, Luminex FlexMap 3D, Illumina NovaSeq 6000
Data analysis	FlowJo (v10), Prism V8 (GraphPad), BD FACSDiva V8, BSMAP 2.90, DSS 2.34, R 3.6.3, minfi 1.32, IGV 2.11.1

For manuscripts utilizing custom algorithms or software that are central to the research but not yet described in published literature, software must be made available to editors and reviewers. We strongly encourage code deposition in a community repository (e.g. GitHub). See the Nature Portfolio guidelines for submitting code & software for further information.

Data

Policy information about availability of data

All manuscripts must include a data availability statement. This statement should provide the following information, where applicable:

- Accession codes, unique identifiers, or web links for publicly available datasets
- A description of any restrictions on data availability
- For clinical datasets or third party data, please ensure that the statement adheres to our policy

Whole genome bisulfite sequencing data was deposited to GEO database under accession number GSE263941. Public available methylation data were downloaded from GEO with accession numbers GSE147667, GSE49031, GSE120878, GSE54719. AML methylation data were downloaded from TCGA data portal with project ID TCGA-LAML. Utokyo T-ALL methylation data were downloaded from NBDC database with accession number JGAS000138. Custom R scripts used for WGBS and methylation array analysis are available upon request.

Research involving human participants, their data, or biological material

Policy information about studies with human participants or human data. See also policy information about sex, gender (identity/presentation), and sexual orientation and race, ethnicity and racism.

Reporting on sex and gender	N/A
Reporting on race, ethnicity, or other socially relevant groupings	N/A
Population characteristics	N/A
Recruitment	N/A
Ethics oversight	N/A

Note that full information on the approval of the study protocol must also be provided in the manuscript.

Field-specific reporting

Please select the one below that is the best fit for your research. If you are not sure, read the appropriate sections before making your selection.

☒ Life sciences ☐ Behavioural & social sciences ☐ Ecological, evolutionary & environmental sciences

For a reference copy of the document with all sections, see nature.com/documents/nr-reporting-summary-flat.pdf

Life sciences study design

All studies must disclose on these points even when the disclosure is negative.

Sample size	The sample size was determined similar to previously published results, see paper references 13, 27, 35.
Data exclusions	No data were excluded from the analyses.
Replication	The experiments have been replicated at least twice.
Randomization	No randomization was used to generate sequencing data.
Blinding	Our measurements/analyses are not subjective so there is no requirement for blind experiments.

Reporting for specific materials, systems and methods

We require information from authors about some types of materials, experimental systems and methods used in many studies. Here, indicate whether each material, system or method listed is relevant to your study. If you are not sure if a list item applies to your research, read the appropriate section before selecting a response.

Materials & experimental systems

n/a	Involved in the study	
☐	☒	Antibodies
☒	☐	Eukaryotic cell lines
☒	☐	Palaeontology and archaeology
☐	☒	Animals and other organisms
☒	☐	Clinical data
☒	☐	Dual use research of concern
☒	☐	Plants

Methods

n/a	Involved in the study	
☒	☐	ChIP-seq
☐	☒	Flow cytometry
☒	☐	MRI-based neuroimaging

Antibodies

Antibodies used	For mouse multi-LT T cells: Purified CD8+ T Cells were then stained with anti-mouse CD8a (53-6.7) from BD Biosciences, anti-mouse CD45.1 (A20), anti-mouse CD45.2 (104), and Ghost Dye from Tonbo Biosciences, and N-Tetramer. CD8a+, VSV-N-Tetramer+, CD45.1 +, CD45.2- cells were sorted on a BD FACS Aria II and 105 sorted cells were transferred via the tail vein into recipient mice. Infections resumed the following day.

	For human CMV specific T cells: After the PBMCs were stained with 0.2 µL NLV-tetramer (NIH Tetramer Core Facility) (CMV pp65; amino acids 495NLVPMVATV503) in 60 µL per well for 25 minutes at room temperature, the PBMCs were incubated with 1.4 µL anti-CD3 FITC (BD Pharmingen, cat. 555339), 2.8 µL anti-CD4 PE/cy7 (Biolegend, cat. 300512), and 2.8 µL anti-CD8 PerCP-cy5.5 (Biolegend, cat. 344710) in 70 µL per well for 30 minutes at4 °C. The PBMCs were washed and the CD3+, CD4-, CD8+, NLV-tetramer+ single cells were isolated by flow cytometric sorting using a FACS Aria II (Becton Dickinson).
Validation	All antibodies are from commercially available sources (see table) and have been validated by the manufactures.

Animals and other research organisms

Policy information about studies involving animals; ARRIVE guidelines recommended for reporting animal research, and Sex and Gender in Research

Laboratory animals	Donor female B6.SJL-PtprcaPepcb/BoyJ (CD45.1+ B6, around 8 weeks old) were bred at the University of Minnesota animal facilities. Female C57BL/6J (CD45.2+ B6, around 8 weeks old) mice were purchased from Jackson Laboratories and served as recipient mice. Aged 2 years old mice were purchased from Jackson Laboratories.
Wild animals	No wild animals were used.
Reporting on sex	Because mouse cells were transferred repeatedly from donor to new recipients, only females were used to prevent rejection.
Field-collected samples	This study did not use field-collected samples.
Ethics oversight	Animals were treated according to the Institutional Animal Care and Use Committee guidelines at the University of Minnesota and St. Jude Children's Research Hospital.

Note that full information on the approval of the study protocol must also be provided in the manuscript.

Plants

Seed stocks	N/A
Novel plant genotypes	N/A
Authentication	N/A

Flow Cytometry

Plots

Confirm that:

- [x] The axis labels state the marker and fluorochrome used (e.g. CD4-FITC).
- [x] The axis scales are clearly visible. Include numbers along axes only for bottom left plot of group (a 'group' is an analysis of identical markers).
- [x] All plots are contour plots with outliers or pseudocolor plots.
- [ ] A numerical value for number of cells or percentage (with statistics) is provided.

Methodology

Sample preparation	For surface staining, samples were washed with and stained in PBS.
Instrument	BD FACS Aria III, BD FACS Canto II, BD FACS Lyric, BD LSRFortessa, BD FACS Aria II
Software	FlowJo (v10) and BD FACSDiva V8
Cell population abundance	Post sort purity >= 90% of total viable cells.
Gating strategy	1) Lymphocytes were identified based on forward and side scatter.<br>2) Singlets: Doublets were excluded using forward and side scatter.<br>3) T cells were defined as CD3+ cells.<br>4) CD4 and CD8 T cells were identified separately.

For Figure 1b, cells were gated:
1)For singlets using FSC-A vs FSC-H and SSC-A vs SSC-H
2)For live cells as live/dead negative
3)For lymphocytes using FSC-A vs SSC-A
4) For CD8a+ using CD8a by CD4
5) For VSV-N Tetramer+ using CD8a by VSV-N Tetramer
6) For transferred 34°, 52°, and recipient cells using CD45.1 by CD45.2

For Figure S4F analysis of mouse T cells, FSC-A vs SSC-A gate was used to identify population targeting lymphocytes. Singlets were separated from doublets by using FSC-A vs FSC-H gating. On the singlets, we selected for live cells using LIVE/DEAE fixable dead cell stain kit (Invitrogen). Among live cell population, we gated CD8 T cells.

For Figure S5D, CD3+, CD4-, CD8+, NLV-tetramer+ single cells were isolated by flow cytometric sorting.

☒ Tick this box to confirm that a figure exemplifying the gating strategy is provided in the Supplementary Information.
